# The Cocaine and Oxycodone Biobanks, Two Repositories from Genetically Diverse and Behaviorally Characterized Rats for the Study of Addiction

**DOI:** 10.1523/ENEURO.0033-21.2021

**Published:** 2021-06-16

**Authors:** Lieselot L. G. Carrette, Giordano de Guglielmo, Marsida Kallupi, Lisa Maturin, Molly Brennan, Brent Boomhower, Dana Conlisk, Sharona Sedighim, Lani Tieu, McKenzie J. Fannon, Nathan Velarde, Angelica R Martinez, Jenni Kononoff, Adam Kimbrough, Sierra Simpson, Lauren C. Smith, Kokila Shankar, Francisco J. Ramirez, Apurva S. Chitre, Bonnie Lin, Oksana Polesskaya, Leah C. Solberg Woods, Abraham A. Palmer, Olivier George

**Affiliations:** 1 Department of Psychiatry, University of California, San Diego, La Jolla, CA 92093; 2 Department of Neuroscience, The Scripps Research Institute, La Jolla, CA 92037; 3 Center for Medical Genetics Ghent, Ghent University, Ghent 9000, Belgium; 4 Department of Internal Medicine, Section on Molecular Medicine, Wake Forest University School of Medicine, Winston-Salem, NC 27157; 5 Institute for Genomic Medicine, University of California, San Diego, La Jolla, CA 92093

**Keywords:** psychostimulant, opioid, substance-related disorders, biological specimen banks, outbred strains

## Abstract

The rat oxycodone and cocaine biobanks contain samples that vary by genotypes (by using genetically diverse genotyped HS rats), phenotypes (by measuring addiction-like behaviors in an advanced SA model), timepoints (samples are collected longitudinally before, during, and after SA, and terminally at three different timepoints in the addiction cycle: intoxication, withdrawal, and abstinence or without exposure to drugs through age-matched naive rats), samples collected (organs, cells, biofluids, feces), preservation (paraformaldehyde-fixed, snap-frozen, or cryopreserved) and application (proteomics, transcriptomics, microbiomics, metabolomics, epigenetics, anatomy, circuitry analysis, biomarker discovery, etc.

Substance use disorders (SUDs) are pervasive in our society and have substantial personal and socioeconomical costs. A critical hurdle in identifying biomarkers and novel targets for medication development is the lack of resources for obtaining biological samples with a detailed behavioral characterization of SUD. Moreover, it is nearly impossible to find longitudinal samples. As part of two ongoing large-scale behavioral genetic studies in heterogeneous stock (HS) rats, we have created two preclinical biobanks using well-validated long access (LgA) models of intravenous cocaine and oxycodone self-administration (SA) and comprehensive characterization of addiction-related behaviors. The genetic diversity in HS rats mimics diversity in the human population and includes individuals that are vulnerable or resilient to compulsive-like responding for cocaine or oxycodone. Longitudinal samples are collected throughout the experiment, before exposure to the drug, during intoxication, acute withdrawal, and protracted abstinence, and include naive, age-matched controls. Samples include, but are not limited to, blood plasma, feces and urine, whole brains, brain slices and punches, kidney, liver, spleen, ovary, testis, and adrenal glands. Three preservation methods (fixed in formaldehyde, snap-frozen, or cryopreserved) are used to facilitate diverse downstream applications such as proteomics, metabolomics, transcriptomics, epigenomics, microbiomics, neuroanatomy, biomarker discovery, and other cellular and molecular approaches. To date, >20,000 samples have been collected from over 1000 unique animals and made available free of charge to non-profit institutions through https://www.cocainebiobank.org/ and https://www.oxycodonebiobank.org/.

## Visual Abstract

**Figure:**
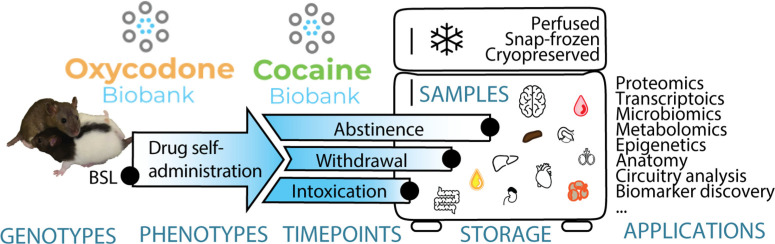

## Significance Statement

The cocaine and oxycodone biobanks offer genetically and behaviorally characterized, longitudinally, and cross-sectionally diverse samples to researchers free of charge. Through this resource, we want to make these valuable samples available to researchers who may not have the equipment or expertise to perform behavioral experiments but can make valuable contributions to the addiction field and facilitate the identification of biomarkers and the development of novel, effective therapies for substance use disorders (SUDs).

## Introduction

Substance use disorders (SUDs) are pervasive in society (Grant et al., 2016; Vadivelu et al., 2018), with dramatic societal and individual consequences. There are currently no FDA-approved pharmacological treatments for cocaine use disorder. While there are FDA-approved treatments for opioid use disorder, the efficacy of these medications varies, and the relapse rate remains high (Eap et al., 2002; de Cid et al., 2008). To improve current treatments and discover new ones, the individual differences in both the propensity to develop addiction-like behaviors (Vowles et al., 2015) and the response to treatment need to be better understood. A critical hurdle in the effort to identify biomarkers and targets is the limited number of repositories that include longitudinal samples, with a detailed characterization of SUD.

To address this, we have created the cocaine biobank (https://www.cocainebiobank.org/) and the oxycodone biobank (https://www.oxycodonebiobank.org/). These repositories contain >20,000 samples from ∼1000 individual rats. Indeed, numerous biological markers have been identified in preclinical models. Failure to replicate or translate such markers to human in the past could be linked to low sample size (usually *N* = 6–12), lack of standard operating protocols between laboratories, and use of inbred strains. Every individual of ∼1000 rats in the biobanks is fully characterized for addiction-like behaviors in a strictly controlled environment using state-of-the-art intravenous self-administration (SA) models with high relevance to SUD. The rats are from the heterogeneous stock (HS). These are highly recombinant animals, established by crossbreeding eight genetically diverse founder strains (Hansen and Spuhler, 1984; STAR Consortium et al., 2008; Baud, 2013; Solberg Woods and Palmer, 2019), resulting in a diversity that mimics the diversity found in the human population. Together with the behavioral characterization, this allows for genome-wide association studies (GWAS) for a variety of addiction-related quantitative traits (Valdar et al., 2006; Baud, 2013; Keele, 2018; Chitre et al., 2020) by whole-genome sequencing of each individual.

Throughout behavioral experiments, samples (feces, urine, blood) are taken longitudinally. Terminal samples (brain, heart, kidneys, liver, colon, ovaries or testis, adrenal glands, and peripheral blood mononuclear cells (PBMCs) are collected from different animals at three timepoints, during (1) intoxication, (2) acute withdrawal, (3) protracted abstinence or from naive rats. To maximize compatibility with a range of downstream applications, samples are (1) perfused using paraformaldehyde, (2) snap-frozen, or (3) cryopreserved. Since all rats are genetically and behaviorally characterized, samples from individuals with specific genotypes or phenotypes can be requested.

The biobanks are cost-effective for the scientific community and created with an open-source mindset (White et al., 2019). Samples are freely available to non-profit organizations. Genetic and behavioral data will be deposited in a public repository like the rat genome database (Smith et al., 2020) or Gene Network (Sloan et al., 2016). Protocols are disclosed here and online (https://www.protocols.io/workspaces/george-lab). The addiction biobanks are particularly useful for researchers from fields outside of addiction, who do not have the resources or expertise to perform chronic intravenous SA. We here introduce the biobanks with methodological details and an example of their application.

## Materials and Methods

Detailed behavioral procedures can be found in the paper’s extended information and the George lab protocol repository on protocols.io (https://www.protocols.io/workspaces/george-lab). All of the procedures were conducted in strict adherence to the National Institutes of Health *Guide for the Care and Use of Laboratory Animals* and were approved by the Institutional Animal Care and Use Committees of The Scripps Research Institute and University of California San Diego.

### Animals

HS rats (sometimes referred to as N/NIH) are provided by Leah Solberg Woods (the only colony in North America). The HS colony was transferred from the Medical College of Wisconsin to the Wake Forest University School of Medicine in 2016. The animals in the current study come from the Wake Forest University School of Medicine colony, which has been named NMcwiWFsm:HS (Rat Genome Database number 13673907). To minimize inbreeding and control genetic drift, the HS rat colony consists of >64 breeder pairs and is maintained using a breeding strategy that takes into account the kinship coefficient of the breeders (Solberg Woods and Palmer, 2019). To maximize genetic diversity, each breeder pair contributes only one male and one female to subsequent cocaine and oxycodone cohorts. The goal is to include 1000 rats in both biobanks consisting of half males, half females. Each rat receives a chip with a radio-frequency identification (RFID) code to keep track of them, their breeding, behavior, organs, and genomic info. Rats are shipped at three to four weeks of age, kept in quarantine for two weeks, and then for the rest of the experiment housed two per cage on a 12/12 h light/dark cycle (lights off at 8:00 A.M. for cocaine, lights off at 4:00 P.M. for oxycodone) in a temperature (20–22°C) and humidity (45–55%) controlled vivarium with *ad libitum* access to tap water and food pellets (PJ Noyes Company).

### Surgery for jugular vein catheterization

Rats are anesthetized with vaporized isoflurane (1–5%). Intravenous catheters are aseptically inserted into the right jugular vein. Catheters consist of Micro-Renathane tubing (18 cm, 0.023-inch inner diameter, 0.037-inch outer diameter; Braintree Scientific) attached to a 90° angle bend guide cannula (Plastics One), embedded in dental acrylic, and anchored with mesh (1 mm thick, 2-cm diameter). Tubing is inserted into the vein following a needle puncture (22 G) and secured with a suture. The guide cannula is punctured through a small incision on the back. The outer part of the cannula is closed off with a plastic seal and metal cover cap, which allows for sterility and protection of the catheter base. Flunixin (2.5 mg/kg, s.c.) is administered as an analgesic and cefazolin (330 mg/kg, i.m.) as an antibiotic. Rats are allowed to recover for a week before any SA. They are monitored and flushed daily with heparinized saline (10 U/ml of heparin sodium; American Pharmaceutical Partners) in 0.9% bacteriostatic sodium chloride (Hospira) that contains 52.4 mg/0.2 ml of cefazolin. Catheter patency is tested (at least at the start and end of the experiment) with brevital sodium (1.5 mg, i.v.), a short-acting barbiturate. Only rats that pass the test (immediate loss of muscle tone) are included in GWAS and the biobanks. In each cohort, a number of rats were not surged and subsequently not exposed to drugs. These naive rats are from the same cohort (same or different breeder pairs) as the drug-exposed animals and thus age-matched control animals.

### Drugs

Oxycodone (Sigma-Aldrich) is dissolved in 0.9% sterile saline (Hospira) and administered at 150 μg/kg per infusion intravenously. Cocaine HCl (National Institute on Drug Abuse, Bethesda, MD) is dissolved in 0.9% sterile saline and administered intravenously at a dose of 0.5 mg/kg per infusion.

### Behavioral testing

All behavioral tests were performed in the dark cycle of the animals. Testing took place while the animals were 7–15 weeks old. Naive rats underwent the behavioral tests that did not require a catheter.

#### Operant SA

SA is performed in operant conditioning chambers (29 × 24 × 19.5 cm; Med Associates) enclosed in lit, sound-attenuating, ventilated environmental cubicles. The front door and back wall of the chambers are constructed of transparent plastic, and the other walls are opaque metal. Each chamber is equipped with two retractable levers that are located on the front panel. Each session is initiated by the extension of two retractable levers into the chamber. Drugs (cocaine, 0.5 mg/kg per infusion in saline or oxycodone, 0.15 mg/kg per infusion in saline) is delivered through plastic catheter tubing connected to an infusion pump. The infusion pump is activated by responses on the right (active) lever that is reinforced on a fixed ratio (FR)1 schedule, with the delivery of 0.1 ml of the drug per lever press over 6 s followed by a 20-s time-out period that is signaled by the illumination of a cue light above the active lever and during which active lever presses do not result in additional infusions. Responses on the left (inactive) lever are recorded but have no scheduled consequences. Fluid delivery and behavioral data recording are computer-controlled. Initially, rats are familiarized and trained to self-administer during ShA sessions (ten sessions of 2 h for cocaine and four sessions of 2 h for oxycodone). The actual SA experiment gives longer access (LgA, 14 sessions of 6 h for cocaine and 12 h for oxycodone). Five sessions are performed per week, with a break over the weekend.

#### Motivation: progressive ratio (PR) responding (both cohorts)

Rats are tested on a PR schedule of reinforcement, in which the response requirements for receiving a single reinforcement increases according to the following equation: [5e(injection number × 0.2)] – 5, which results in: 1, 2, 4, 6, 9, 12, 15, 20, 25, 32, 40, 50, 62, 77, 95, 118, 145, 178, … for cocaine and 1, 1, 2, 2, 3, 3, 4, 4, 5, 5, 6, 6, 7, 7, 8, 8, 9, 9, 10, 10, 11, 12, 13, 14, … for oxycodone. The break point is defined as the last ratio attained by the rat before a 60-min period during which a ratio is not completed, at which point the program automatically terminates. PR is performed after ShA and LgA, besides after footshock in the cocaine cohorts.

#### Irritability-like behavior: bottle brush test (cocaine cohorts only)

Testing consists of ten 10-s trials with 10-s intertrial intervals in plastic cages (27 × 48 × 20 cm) with clean bedding. The rat is placed in the back of the cage, and a bottlebrush was rotated rapidly toward its whiskers. Three trained observers record in real-time both aggressive responses (smelling, biting, boxing, following, and exploring the bottlebrush) and defensive responses (escaping, digging, jumping, climbing, defecation, vocalization, and grooming). Total aggressive and defensive scores are calculated for each animal based on the average score of the observers. Both aggressive and defensive behaviors are summed to calculate the total irritability score. Irritability-like behavior reflects a composite measure of aggressive versus defensive responses. The test is performed after surgery (baseline) and after LgA 18 h in withdrawal.

#### Mechanical nociception: von Frey test (oxycodone cohorts only)

Hind paw withdrawal thresholds (time and force) are determined 12 h into withdrawal using a dynamic plantar aesthesiometer for mechanical stimulation (electronic Von Frey, Ugo Basile), which automates Von Frey measurements. The needle of the device is placed to touch under the back paw of the rat (who sits in a box on a metal grid), and then the force increases at a constant rate (from 0 to 40 in 20 s) until the rat lifts its paw. Time and force for paw lifting are recorded. The measurement is repeated 3× for each hind paw and averaged. The test is performed before surgery (baseline) and in withdrawal 12 h after the last LgA session.

#### Compulsive-like responding: contingent footshock (cocaine cohorts only)

During this FR1 SA protocol, 30% of active lever presses are randomly associated with a footshock (0.3 mA for 0.5 s) through the cage’s floor grid. This test is only performed in the cocaine cohort after LgA, as the analgesic effect op opioids could confound this measure.

#### Opioid-induced analgesia: tail immersion test (oxycodone cohorts only)

The rat is secured in a towel and its tail tip immersed in hot water (52°C). The latency to lift the tail out of the water is recorded. This test is performed before surgery without any opioid administration (baseline), and after administering oxycodone (0.15 mg/kg, i.v.) both before the start of the first ShA session and before the last LgA session to evaluate the development of tolerance to the analgesic effect of oxycodone during SA.

#### Medication testing (oxycodone cohorts only)

Rats are treated with intraperitoneal injections of buprenorphine (0.5 mg/kg), methadone (3 mg/kg), naloxone (3 mg/kg), and vehicle 30 min before a PR session using a within-subject Latin square design. The drug test days are separated by a LgA session without treatment.

### Biological sampling

All samples are stored at −80°C.

#### Longitudinal samples

##### Fecal pellet collection

Feces (two droppings on average) are collected fresh from the animal in a sterile tube by gently massaging the rectal canal and snap-frozen on dry ice. Fecal pellet collection is performed before surgery (baseline), after short access (ShA), and after long access (LgA). For cocaine: post-ShA feces are collected right after the session finished (intoxication), while post-LgA feces are collected 18–24 h after finishing the session (withdrawal). A limited set of feces was also collected in abstinence. For oxycodone: all feces are collected in withdrawal, 16–18 h for ShA and 7–8 h for LgA.

##### Blood collection

While the rats are anesthetized for the intravenous catheter surgery, 200- to 400-μl blood (baseline) is collected through retroorbital bleed in EDTA-coated tubes that are immediately inverted five times. Blood samples are processed within 1 h of collection by centrifugation at 2000 × *g* at room temperature (RT) for 10 min to pellet the erythrocytes. The supernatant plasma is immediately transferred into a fresh tube, scored for quality on a scale from 0 to 5, snap-frozen on dry ice, and stored at –80°C.

##### Urine collection

Urine (>100 μl) is collected from rats isolated overnight on a grid over non-absorbent sand at baseline and in acute withdrawal after LgA.

##### Vaginal smear

Cells from the vaginal opening of female rats are collected with a wet cotton Q swab and dabbed onto a glass microscope slide to determine where the females are in their estrous cycle (proestrus, estrus, metestrus, or diestrus) at baseline, after the last ShA and LgA, and before dissection.

#### Terminal samples

After the last LgA session and completing the behavioral characterization, rats are killed at three different timepoints. (1) Intoxication: euthanasia is performed during the last SA session (after ∼2 h of drug access). (2) Acute withdrawal: euthanasia is performed 18 h (for cocaine) and 12 h (for oxycodone) after the end of the last SA session. (3) Protracted abstinence: euthanasia is performed four to five weeks after the last SA session. The age of animals at killing is ∼16–18 weeks for the intoxication and acute withdrawal timepoints and 21–23 weeks for the protracted abstinence time point. For quality control, audio recordings are made during the dissections. Rats are anesthetized with CO_2_, followed by the following.

**Figure 1.**
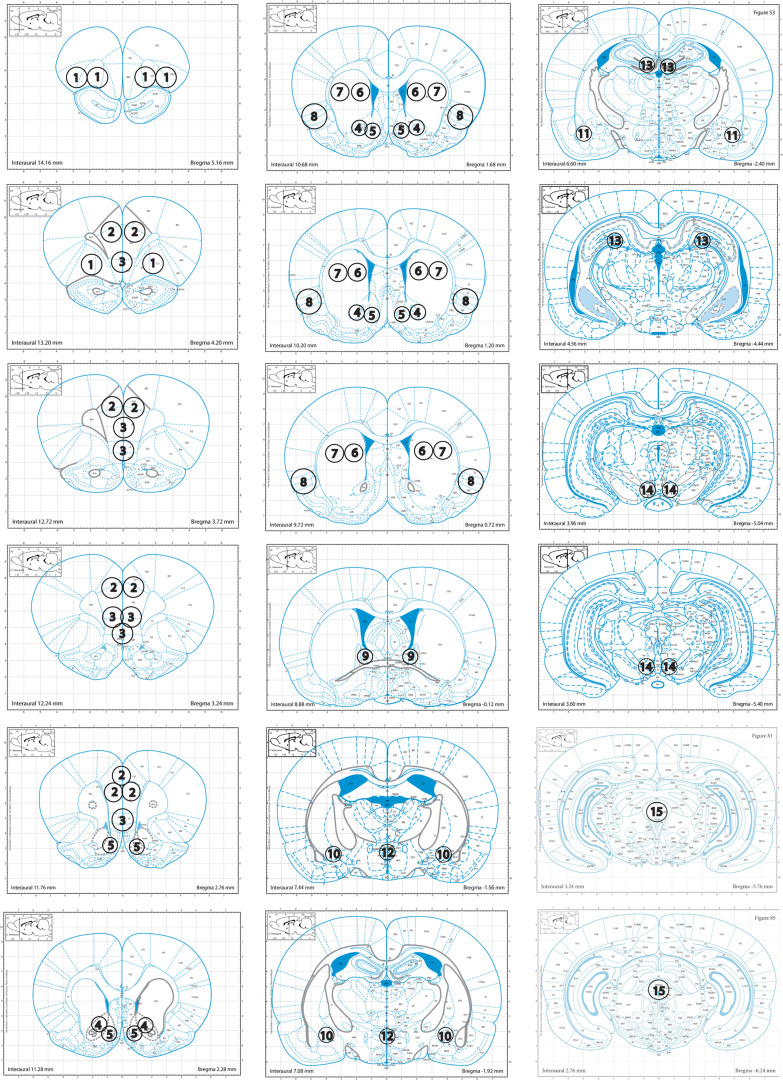
Punch maps for 1 = orbitofrontal cortex (OFC); 2 = dorsal and 3 = ventral prefrontal cortex (dPFC, vPFC); 4 = core and 5 = shell of the nucleus accumbens (NAc); 6 = dorsal medial and 7 = dorsal lateral striatum (DMS, DLS); 8 = insular cortex (insula); 9 = dorsal bed nucleus of the stria terminalis (dBNST); 10 = central nucleus of the amygdala (CeA); 11 = basolateral amygdala (BLA); 12 = paraventricular nucleus (PVN); 13 = dentate gyrus of the dorsal hippocampus (DG); 14 = ventral tegmental area (VTA); 15 = periaqueductal gray (PAG), modified from *The Rat Brain in Stereotaxic Coordinates*, Ed 5, by (Paxinos and Watson, 2004).

 (1) Blood collection (∼8 ml) via cardiac puncture in EDTA coated tubes that are immediately inverted five times. For plasma, up to 3 ml of blood is transferred to sterile Eppendorf tubes, processed as above, and snap-frozen in two aliquots of ∼0.5 ml. PBMCs are also prepared from the blood in a tissue culture hood. First, whole blood (2 ml) is diluted with an equal volume (2 ml) of freshly prepared Tris-buffered saline (TBS; 10 mm, pH 7) with 2% fetal bovine serum (FBS). This diluted blood (4 ml) is then carefully layered over 2 ml of density barrier (TBS diluted in H_2_O at 2.5:0.5 mixed with OptiPrep in a 2.7:9.3 ratio, to achieve a final osmolarity of 242 ± 10 mOsm) in a 12 ml Falcon tube. Tubes are centrifuged at 700 × *g*, RT for 30 min with slow acceleration and deceleration to prevent disruption of the interface. After separation, PBMCs can be collected and diluted in an equal amount (∼200 μl) of TBS with 2% FBS. Cells are pelleted by centrifugation at 400 × *g*, RT for 10 min. The pellet is scored for quantity on a scale from 0 to 3, resuspended in 250 μl Cytostor10 (Stemcell Technologies), transferred into a cryotube, and slowly frozen to –80°C.

(2) Brains are extracted from the skull and submerged in a slurry of 2-methylbutane with dry ice (at –30°C) for about 8 s, till fully frozen. Brains are put in a container on dry ice till final storage at –80°C. Punches from selected regions are collected from 500-μm brain slices according to the maps in Figure 1. They are kept frozen at all times and stored at –80°C.

(3) Other organs. Heart, liver (left and right median lobe), kidney (left and right), adrenal gland (left and right), ovary (left and right), or testicles (left and right) are dissected out, rinsed in PBS, weighed, and snap-frozen in their respective containers on dry ice. Colon, cecum + poop, and tail tip are dissected and snap-frozen in their respective containers on dry ice. The dissected spleen is combined with the RFID chip in PBS in its tube that is snap-frozen on dry ice. The spleen is used for genotyping. The tail tip (3/4”) is stored as back-up material for that purpose.

Alternatively, rats are killed under anesthesia by exsanguination (blood was collected from the trunk and further processed as described above) and perfusion with 100 ml of PBS followed by 400 ml of 4% paraformaldehyde. The brains and other organs are then postfixed in paraformaldehyde overnight and transferred to 30% sucrose in PBS/0.1% azide solution at 4°C for 2–3 d, before freezing and storing at −80°C.

### Data analysis

Z-scores are used to reduce sex and cohort effects and scale the different behavioral paradigm outputs. They are calculated as follows Z = (x-μ)/σ, where x is the raw value, μ is the mean of the cohort, and σ is the standard deviation of the cohort. Z-scores are calculated for the following measures:

Escalation index: E = Z_f_/Z_0_, where Z_f_ is the Z-score of an animal on the last 3 d of escalation and Z_0_ is the Z-score of an animal during the first day of escalation. A daily Escalation Index (E_i_) can be calculated for each session (E_i_ = Z_i_/Z_0_), where Z_i_ is the Z-score on a given escalation session i. This index can also be calculated for the first hour or the entire session. Pain index (withdrawal): Z-scores are calculated from the pain threshold in withdrawal as a percentage of the baseline threshold.Irritability index (withdrawal): Z-score of the total irritability score with the baseline scores subtracted. PR index (motivation): Z-score of the breakpoint after LgA. Shock index (compulsivity): Z-score of the number of infusions. Tolerance index (intoxication): Z-scores are calculated from the difference in latency to lift the tail from a hot water bath after a dose of oxycodone between the end of LgA and before the first ShA session.

The addiction index (AI) is obtained by averaging relevant behavioral indexes.

### Results

### Characterization of addiction-like behaviors

**Figure 2.**
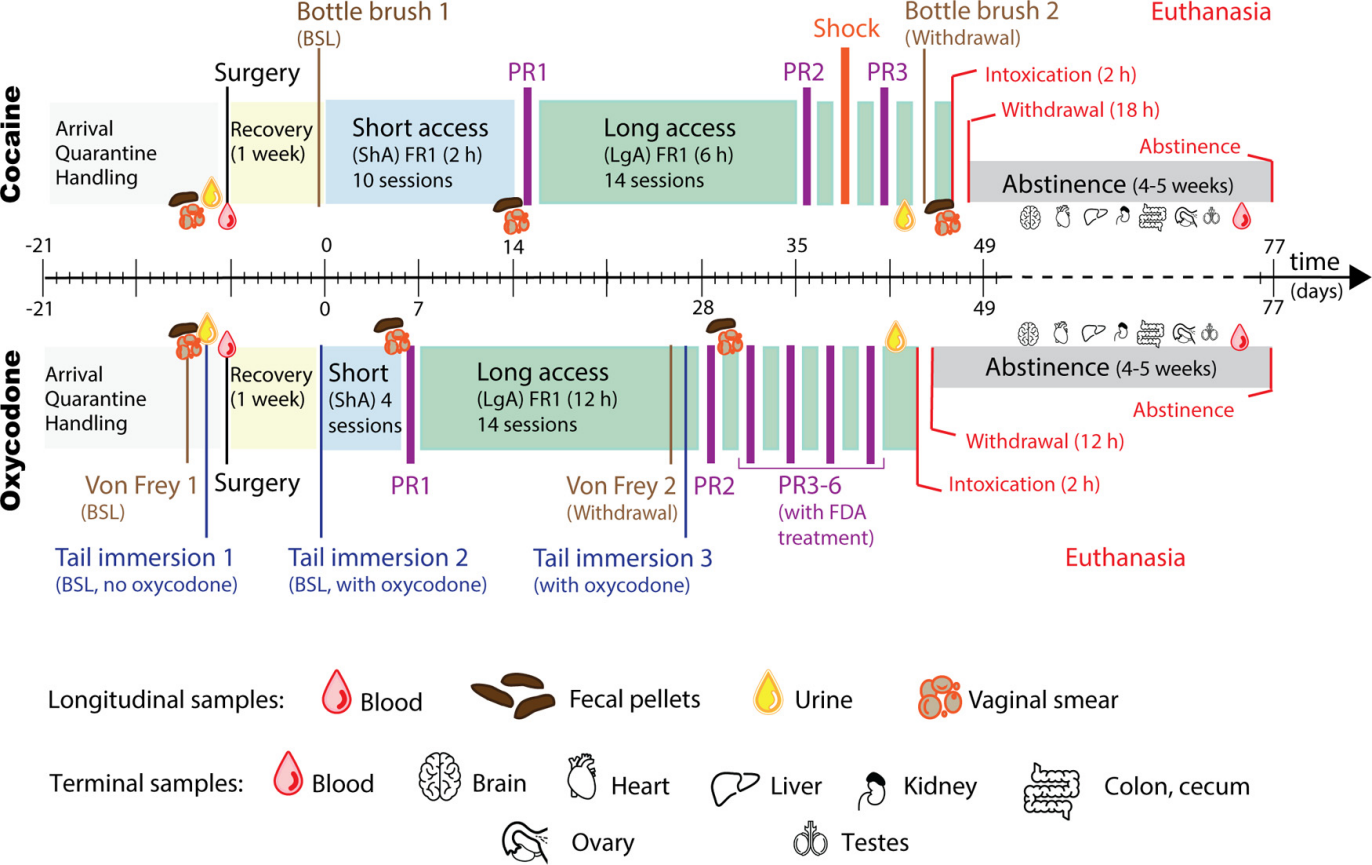
Experimental design of addiction-like behavior phenotyping and sampling. Timeline of behavioral tests and biological sampling relative to drug SA. Testing and sampling were performed on 6- to 15-week-old rats at baseline (BSL; before drug exposure and surgery), after ShA, and LgA to the drug, as well as at euthanasia either during intoxication, acute withdrawal (16–18 weeks old) or after prolonged abstinence (21–23 weeks old). The timeline starts with three- to four-week-old rats (shipped from WFU and quarantined at TSRI or UCSD for two weeks). The longitudinal samples collected include blood, feces, urine, and vaginal smears. The terminal samples include brain, heart, liver, kidney, colon, cecum, adrenal glands, ovaries, and testicles. The behavioral measures include hyperalgesia using the von Frey test (for oxycodone) at LgA compared with BSL, irritability-like behavior using the bottlebrush test (for cocaine) at LgA compared with BSL, opioid-induced analgesia, and tolerance using the tail immersion test (for oxycodone) before ShA and after LgA with oxycodone on board relatively compared with BSL and BSL with oxycodone, motivation with PR test (for both oxycodone and cocaine) after ShA and LgA (and after shock for cocaine only), compulsivity with FR1 using contingent footshocks (for cocaine) and the effect of FDA-approved treatments after LgA on PR tests (for oxycodone).

The timeline of our standard protocols for both cocaine and oxycodone addiction-like behavior characterization is shown in Figure 2. Following surgery and one week of recovery, rats are given ShA to the drug for training (10 × 2-h sessions for cocaine at 500 μg/kg per infusion and 4 × 2-h sessions for oxycodone at 150 μg/kg per infusion). Then 14 d of LgA drug SA followed (6-h sessions for cocaine and 12-h sessions for oxycodone; same doses). Longitudinal biological samples including blood, urine, and feces are collected at three timepoints during behavioral testing: (1) at BSL before drug exposure; (2) after ShA (only feces) and; (3) after LgA (Table 1).

**Table 1:** Summary of the composition of the biobank samples, giving the number of animals per sample, grouped by drug, time point, and processing per sample type

Number of animals		Cocaine			Oxycodone			Number of samples per animal
Longitudinal	Sample	BSL	ShA	LgA	BSL	ShA	LgA	
	Urine	418	-	360	421	-	351	1 per timepoint
	Feces	714	418	628	421	273	285	1 per timepoint
	Plasma	418	-	Terminal	421	-	Terminal	1 per timepoint

Terminal	Sample	Intoxication	Withdrawal	Abstinence	Intoxication	Withdrawal	Abstinence	
	Brain	167/36 (snap/PFA)	160/36 (snap/PFA)	199/21 (snap/PFA)	82 (snap)	85 (snap)	184 (snap)	1–15 (whole brain or ∼15 regions)
	Heart	150/36 (snap/PFA)	144/36 (snap/PFA)	125/21 (snap/PFA)	82 (snap)	85 (snap)	78 (snap)	1
	Liver	150/36 (snap/PFA)	144/36 (snap/PFA)	125/21 (snap/PFA)	82 (snap)	85 (snap)	78 (snap)	2 (right, left)
	Kidney	150/36 (snap/PFA)	144/36 (snap/PFA)	125/21 (snap/PFA)	82 (snap)	85 (snap)	78 (snap)	2 (right, left)
	Adrenal Gland	150/36 (snap/PFA)	144/36 (snap/PFA)	125/21 (snap/PFA)	82 (snap)	85 (snap)	78 (snap)	2 (right, left)
	Ovary	72/18 (snap/PFA)	66/17 (snap/PFA)	60/12 (snap/PFA)	43 (snap)	40 (snap)	38 (snap)	2 (right, left)
	Testes	78/18 (snap/PFA)	78/19 (snap/PFA)	65/9 (snap/PFA)	39 (snap)	45 (snap)	40 (snap)	2 (right, left)
	Colon	150/36 (snap/PFA)	144/36 (snap/PFA)	125/21 (snap/PFA)	82 (snap)	85 (snap)	78 (snap)	1
	Cecum	150/36 (snap/PFA)	144/36 (snap/PFA)	125/21 (snap/PFA)	82 (snap)	85 (snap)	78 (snap)	1
	Plasma	203 (snap)	196 (snap)	147 (snap)	82 (snap)	85 (snap)	106 (snap)	2 aliquots
	PBMCs	158 (snap)	151 (snap)	101 (snap)	-	-	-	1

Samples from naive animals are included in the given number and represent ∼20% of samples. Snap, snap-frozen; PFA, paraformaldehyde.

During ShA and LgA, which follow a FR1 schedule of reinforcement, drug intake is measured. An essential step in the characterization of addiction-like behaviors is determining the escalation of drug intake. While some rats escalate, others show no escalation or even decrease intake. Importantly, HS rats exhibit high interindividual differences and low intraindividual variability. Also important are motivation and compulsive-responding for drug, evaluated respectively using an exponential increase in cost with a PR schedule (after ShA and LgA) and an adverse consequence (contingent footshock after LgA; for cocaine; Deroche-Gamonet et al., 2004; Chen et al., 2013). Withdrawal severity is evaluated by irritability-like behavior (bottlebrush test after LgA compared with BSL; for cocaine; Kimbrough et al., 2017) and hyperalgesia/allodynia (von Frey test after LgA compared with BSL; for oxycodone; Chaplan et al., 1994). For oxycodone, analgesia and tolerance are evaluated (tail immersion test) in addition to the effects of FDA-approved treatments (buprenorphine, methadone, and naltrexone with PR responding).

**Figure 3.**
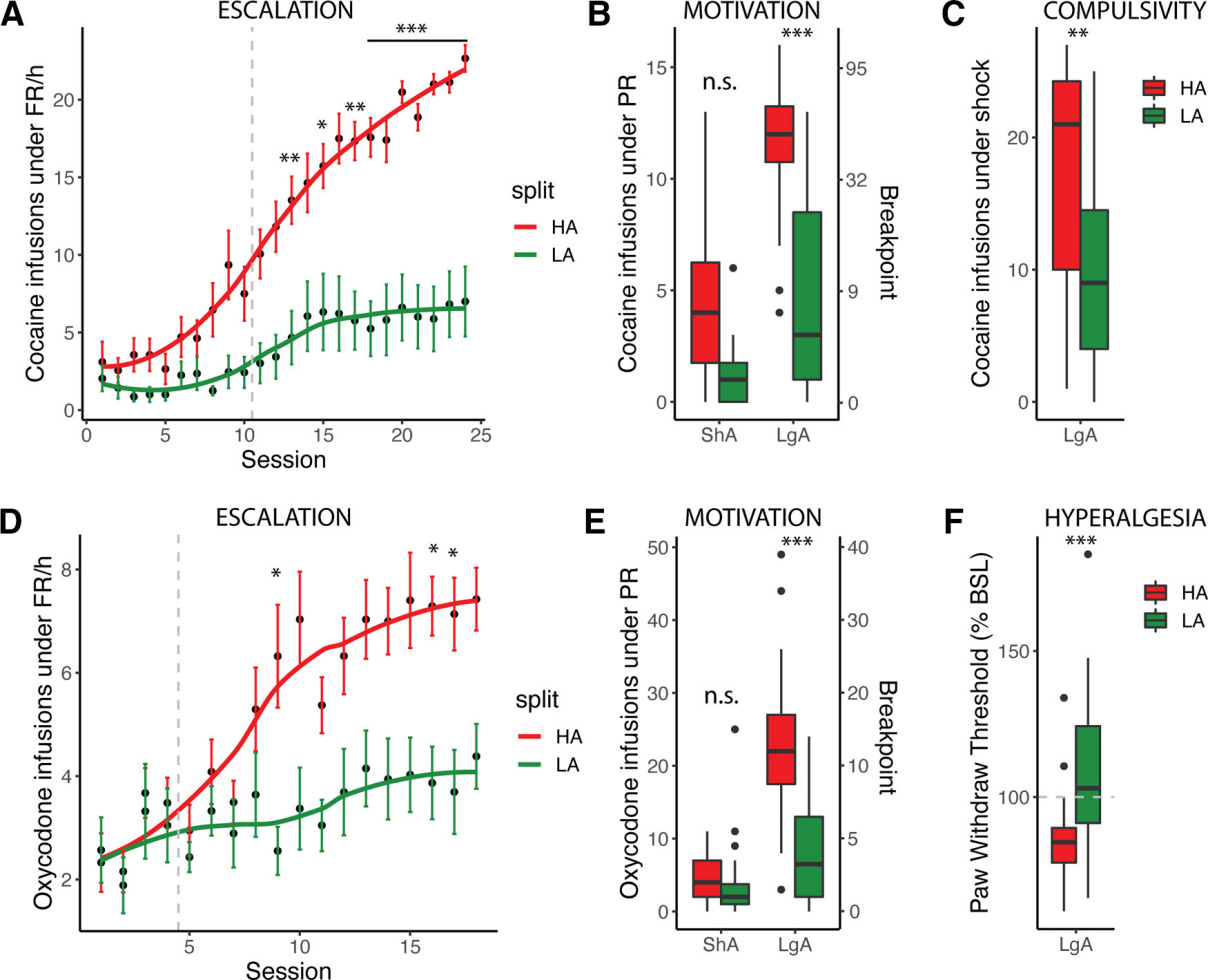
Characterization of addiction-like behavior with the AI. Example of addiction-like behaviors assessed in rats and used for the calculation of the AI: escalation (***A***), motivation (***B***), and compulsivity (***C***) for cocaine (*N* = 39, cohort 04) or escalation (***D***), motivation (***E***), and hyperalgesia (***F***) for oxycodone (*N* = 52, cohort 06). The data are represented following a median split of the cohort’s AI with animals with high addiction-like behaviors (HA) in red and low addiction-like behaviors (LA) in green. Error bars represent the standard error of the mean (***A***, ***D***). Boxplots represent the median with the 75% quartile (upper hinge), 25% quartile (lower hinge), and observations smaller than or equal to upper hinge + 1.5 * interquartile range (IQR; upper whisker) or greater than or equal to lower hinge – 1.5 * IQR (lower whisker) (***B***, ***C***, ***E***, ***F***). Data beyond the end of the whiskers (“outlying” points) are plotted individually. Statistics represent the difference between HA and LA [*p < 0.05, **p < 0.02, ***p < 0.001, not significant (n.s.) and were obtained with two-way ANOVA for each behavioral measure, using Bonferroni’s multiple comparison test where appropriate].

Animals can be characterized based on individual behavioral measures or a composite AI as adapted from Deroche-Gamonet et al. (2004) and (Belin et al. (2009), that classifies them as vulnerable (high addiction-like behavior; HA) with high AI versus resilient (low addiction-like behavior; LA) with low AI by a median-split. For cocaine the AI is the average of the Z-scores of escalation (intake during LgA FR1), motivation (intake during PR) and compulsivity (intake despite footshock). For oxycodone, the Z-scores of escalation, motivation, and hyperalgesia (reduced pain threshold during withdrawal) are averaged. Figure 3 shows the raw data for these behavioral measures for HA and LA obtained from one cohort each.

### Currently available samples in the biobanks

Terminal samples (e.g., brains and other organs) are available from different rats at three timepoints (Fig. 2; Table 1): (1) after 2 h of SA, to study the effect of drug intoxication; (2) after 12 h (for oxycodone) or 18 h (for cocaine) past the end of the last SA session to study the effect of withdrawal; (3) after four to five weeks of protracted abstinence in the home cage to evaluate long-lasting biological changes (Garavan et al., 2013). The animals are assigned to each time point in a pseudo-randomized fashion to ensure a representative distribution of HA and LA rats. Additionally, we maintain a group of age-matched control animals that are not exposed to drug.

For maximal compatibility with the widest variety of potential experiments, samples are preserved with or without fixation. Traditional histology and anatomy studies do better on perfused tissues that are cleared of blood and crosslinked with paraformaldehyde (Gage et al., 2012) because this preserves the anatomic organization of the tissue and has a very long shelf-life (>10 years). However, those crosslinks disturb many molecular biology protocols, which work better with fresh, rapidly frozen tissue (snap-frozen; Auer et al., 2014). Alternatively, structurally intact living cells are cryopreserved to keep cells viable with a long shelf-life. PBMCs are extracted from whole blood and slowly frozen with cryoprotectant (Rasooly et al., 2017). PBMCs can be used to prepare induced pluripotent stem cells (iPSCs) and thus virtually any other cell type (Takahashi and Yamanaka, 2006; Hokayem et al., 2016).

The two biobanks currently have over 20,000 samples from ∼1,100 unique animals, aiming to grow to double in size. Brains can be requested as a whole, sliced, or punched from at least 15 different brain regions (Fig. 1). All samples are stored at −80°C in multiple freezers, located in different buildings with back-up generators to limit loss in case of adverse events. Freezer temperatures are monitored online with an alarm and notification service (Minus80 monitoring; https://minus80monitoring.com/).

### The biobank ecosystem

The biobanks are currently collaborating on 27 independent research projects with major universities and research institutes in the United States and Europe, to which we have shipped out >1000 samples (Table 2). Current and past collaborative projects have used brain tissue for circuitry analysis, histology, proteomics, epigenetic analysis, and single-cell sequencing. Plasma is being used for exosome transcriptomics, metabolomics, and biomarker discovery. Feces have been analyzed in microbiome projects and for biomarker discovery. Pelvic floor muscle and semen were dissected by special request to study oxycodone’s and cocaine’s effect on their composition, respectively. Publications that result from the use of biobank samples will be tracked on the websites (https://www.cocainebiobank.org/ and https://www.oxycodonebiobank.org/).

**Table 2:** Examples of ongoing collaborations with samples from the biobanks

Sample	Preservation	Application
Brain	Snap-frozen	Proteomics
Brain	Perfused	Circuitry analysis
Brain	Perfused	Histology and cellular activation
Brain (+punches)	Snap-frozen	Epigenetics and epigenomics
Brain (+punches)	Snap-frozen	Single-cell sequencing
Semen	Snap-frozen	Semen composition
Muscles	Snap-frozen + OCT embedded	Muscle composition
Feces	Snap-frozen	Microbiome analysis
Feces	Snap-frozen	Biomarker discovery
Plasma	Snap-frozen	Metabolomics
Plasma	Snap-frozen	Biomarker discovery
Plasma	Snap-frozen	Exosome transcriptomics

**Figure 4.**
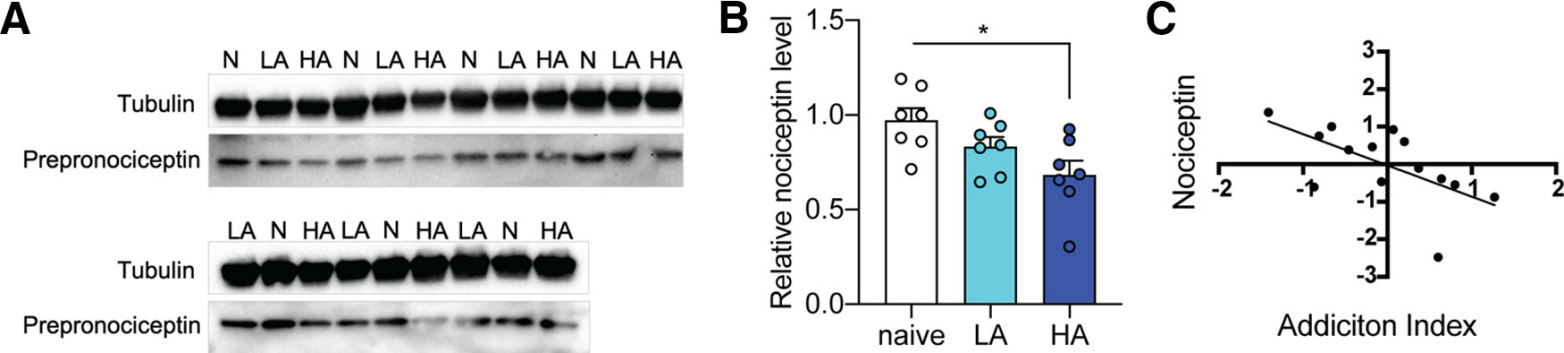
Application example looking at N/OFQ levels in oxycodone biobank brain punches from rats with different addiction profiles. ***A***, Immunoluminescent Western blottings that show nociceptin levels in naive, LA, and HA rats; *n* = 21 (*n* = 7 per group). ***B***, Western blot analysis revealed a significant decrease in nociceptin levels (internally normalized to β-tubulin and a naive rat on the blot) in HA rats compared with naive rats; *p = 0.02 one-way ANOVA, error bars represent standard error of mean. ***C***, Correlation between AI and the Z-score of nociceptin levels in the CeA; *p < 0.02 pearson correlation. Partial figure reproduced from Kallupi et al. (2020).

An application example was recently published (Kallupi et al., 2020). Kallupi and colleagues were interested in investigating nociceptin/orphanin FQ peptide (N/OFQ, nociceptin) levels in the CeA of rats with high versus low addiction profiles for oxycodone to confirm a preliminary hypothesis. N/OFQ is an endogenous opioid-like peptide with an important role in opioid tolerance and reward (Ciccocioppo et al., 2000). Analysis of CeA punches from the oxycodone biobank selected with a high (HA, *N* = 7) or low (LA, *N* = 7) addiction phenotype based on their AI and naive controls (*N* = 7) by Western blot analysis (Fig. 4*A*) showed a significant reduction of the peptide in HA rats compared with naive rats (one-way ANOVA: *p* = 0.02, *post hoc*: *p* = 0.02; Fig. 4*B*). Moreover, individual N/OFQ levels correlated with the animals’ AI (*r* = −0.62, *p* = 0.017; Fig. 4*C*). These results confirmed that the N/OFQ levels in the CeA inversely correlate with addiction-like behaviors and illustrate these genetically-diverse samples’ utility.

## Discussion

This report describes the cocaine and oxycodone biobanks, two biological repositories aimed at facilitating research on the biological mechanisms underlying addiction. Every sample in the biobanks can be selected based on phenotype or genotype and comes with a comprehensive report of our behavioral and genetic characterization. The report also includes the animal identification number, sex, age, body weight, the weight of the sample, collection date, and time from anoxia to freezing. Samples are available both longitudinally and cross-sectionally, tracked using RFID chips and barcodes to ensure tracing and transparency. Longitudinal samples currently include blood plasma, feces, and urine collected at baseline, after ShA, and LgA (Fig. 2; Table 1). Organs and other terminal samples are harvested during drug intoxication, acute withdrawal, or protracted abstinence or from naive controls (Fig. 2; Table 1). Ongoing collaborations and other potential applications include omics-studies and neuroanatomy, biomarker discovery, and other cellular and molecular approaches (Table 2). To maximize compatibility with this variety of downstream applications, samples are cryopreserved, fixed, or snap-frozen.

Addiction-like behavior is characterized using multiple behavioral paradigms with standardized operating procedures (SOPs). The animal model of LgA to drug SA has shown high face, predictive/postdictive, and construct validity and is thus highly relevant to psychostimulant and opioid use disorders (George et al., 2008, 2014; Vendruscolo et al., 2011; Edwards and Koob, 2013; Wade et al., 2015). It is associated with neuroadaptations that are also observed in humans with cocaine use disorder (Briand et al., 2008; George et al., 2008). The model has been shown to exhibit at least seven of the 11 criteria for SUD listed in the DSM-5, including most of the criteria required to diagnose severe use disorder: (1) tolerance (Ben-shahar et al., 2006), (2) withdrawal (Ahmed et al., 2002; Vendruscolo et al., 2011), (3) substance taken in larger amount than intended (Ahmed and Koob, 1998), (4) unsuccessful efforts to quit (Ahmed and Cador, 2006), (5) considerable time spent to obtain the drug (Wee et al., 2008), (6) important social, work, or recreational activities given up because of use (George et al., 2008; Lenoir et al., 2013), and (7) continued use despite adverse consequences (Vanderschuren and Everitt, 2004; Xue et al., 2012). Sex differences in cocaine and oxycodone intake have been identified using this model, as seen in human, with females appearing more vulnerable (Roth and Carroll, 2004; Ramôa et al., 2013; Kimbrough et al., 2020). Behavioral testing for the Biobanks confirmed these findings with significant differences in escalation, intake, motivation and compulsivity. Samples can be selected based on sex and estrous cycle to facilitate research into these differences. Moreover, while the LgA model was only used by a few laboratories a decade ago, it has now been widely adopted in the field and is routinely used in prominent laboratories in North America and Europe. A major benefit of the biobanks, and SOPs, is the potential to reduce often-observed nonspecific behavioral variability between studies because of differences in operant training, learning, drug priming, stress, food restriction, and circadian cycle.

Family and twin studies demonstrate that ∼50% of the vulnerability to cocaine and opioid use disorders is determined by genetic factors (Kreek et al., 2005; Agrawal et al., 2012; Ducci and Goldman, 2012). Recently, large human GWAS have begun to identify some of the genes involved in SUDs (Walters et al., 2018; Kranzler et al., 2019; Liu et al., 2019; Sanchez-Roige et al., 2019a,b, 2020; Erzurumluoglu et al., 2020; Polimanti et al., 2020; Zhou et al., 2020a,b); however, the vast majority of genes that confer risk remain unknown. Therefore, the genes/alleles that mediate individual differences in the effect of cocaine and opioids and the biological mechanisms responsible for developing addiction-like behaviors are still poorly defined (Klepstad et al., 2011; Hart et al., 2013). Taking advantage of the fact that the eight founders of the HS rats have been sequenced, HS rats can be used to map genetic loci to one or a few Mb. Whole-genome sequencing identifies hundreds of thousands of single nucleotide polymorphisms (SNPs) per individual and, following imputation, provides genotypes at millions of SNPs. Combining hundreds of animals’ genetic and genomic data will feed a subsequent GWAS to identify gene variants contributing to vulnerability and resilience to SUD-like behavior (Chitre et al., 2020), which forms the basis to identify specific genetic loci, estimate heritability, investigate genetic correlations and perform phenome-wide association studies (PheWAS) and transcriptome-wide association studies (TWAS). All of which may identify novel gene variants to inform human personalized diagnosis and treatment. Further expansion of the biobank ecosystem also aims to facilitate the integration of genetic complexity studies into other fields of addiction neuroscience.

Certain difficulties associated with clinical studies, including imprecision of self-reported drug addiction measures, the lack of longitudinal analyses of behavior, lack of biological samples, the lack of control of environmental variables, and qualitative versus quantitative measures, can be overcome through the use of animal models. Preclinical settings allow for longitudinal analyses with qualitative and quantitative measures that can be highly standardized and replicated while minimizing the variability because of the environment. Such a strategy has the potential to reveal a greater number of gene variants, metabolites, protein regulations, and neuroadaptations associated with the vulnerability to develop compulsive cocaine or oxycodone use. Moreover, because we have so many animals, these repositories will facilitate replication, validation, and follow-up studies that are virtually impossible in human subjects and extremely difficult in regular small-size [at least one or two order(s) of magnitude lower than current] preclinical studies. Despite the large number of animals in the biobank, grouping the samples in a biobank helps reduce the total number of lab animals and is thus beneficial for animal welfare. Such a resource is also cost-effective to the scientific community and will facilitate addiction research by making samples available to researchers who do not have the capability to perform advanced behavioral and genetic analysis.

## Synthesis

Reviewing Editor: Alexxai Kravitz, Washington University in St. Louis Decisions are customarily a result of the Reviewing Editor and the peer reviewers coming together and discussing their recommendations until a consensus is reached. When revisions are invited, a fact-based synthesis statement explaining their decision and outlining what is needed to prepare a revision will be listed below. The following reviewer(s) agreed to reveal their identity: Megan Fox.

Reviewer consensus statement of essential comments:

1) The authors should clarify their reporting of the behavioral studies performed on the animals included in the Biobank. Figure 1A was confusing to the reviewers and would benefit from breaking the schematic into separate panels for the different behavioral tests that were run on different cohorts. Since behavioral characterizations also differed between the oxy and cocaine animals (along with the number of ShA training sessions, etc) it would be useful to create a separate panel for each drug. Figure 1A could also be combined/integrated with Figure 2, instead of with Figure 1B (see comment 2).

2) Regarding Figure 1B, it would be helpful if this figure contained a summary of raw data/info on the animals in the Biobank. Researchers are likely to want to know the demographics (age, sex) of animals in the Biobank, as well as levels of lever-pressing and/or amount of drug intake that was achieved by animals in the Biobank, and not just the z-scores of their responding relative to the mean. Reporting variance in drug intake could also assist readers in understanding their ability to obtain data from escalators vs. non-escalators from the Biobank. While some of this information is in Table 1, a graphical summary would be more useful than the present data shown in Figure 1B.

In addition, it appears that data from cocaine and oxycodone self-administering rats were combined together in Figure 1B. As these drugs are of different classes and the addiction index is calculated from different behaviors, it is necessary to show data sets from each drug separately. Finally, the figure should employ a legend for what the light and dark blue bars represent. Breaking this analysis apart from Figure 1A and expanding it into its own figure may be a clearer way to present the information.

3) It is not clear whether “age-matched control animals” (line 206) refer to littermates of the drug-exposed animals, or if these are from separate breeding pairs. Please clarify.

4) There were several methodological details that were omitted from this manuscript and should be reported. See full reviewer comments on these below.

5) A graphical abstract could enhance this paper.

*******************************

Full comments from Reviewer #1:

*******************************

This manuscript has the potential to advance the field if more information about the recorded behaviors are provided by the authors in a revision. These collected samples are targeted for use in laboratories that would not otherwise be able to generate similar tissue sets. Additionally, as the authors ran both cocaine and oxycodone IV self-administration to produce comparable biobanks, samples from these biobanks have the potential to confer meaningful data about parallels between the two classes of drugs of abuse. However, as presented, this manuscript itself does not provide novel data to advance the field.

Major:

This manuscript introduces two biobanks that aim to expand the accessibility of tissue obtained following rat drug self-administration, which could benefit many researchers. However, as the two data panels (Figure 1b and Figure 3) presented are already published figures, the manuscript does not currently offer any novel data. As the authors have done a significant amount of work characterizing a variety of drug-related behaviors, displaying these data would allow for better transparency and provide further validity to the banked samples. There are many approaches that would achieve this point. For example:

- Providing data related to average intake or responding across FR1 sessions is important for understanding the level of drug intake that was achieved in these studies. The authors could also replicate known effects, such as sex differences in intake/motivation.

- Another benefit mentioned by the authors is the ability to detect individual differences. Examples of this from the recorded behaviors would greatly underscore the authors' points. For example, displaying data for escalators vs non-escalators in cocaine and oxycodone groups would supplement the authors' statements in the Results section.

- Additionally, because the authors split rats into low and high addiction-like groups and state that this characterization is a major benefit of their biobank, percentages of rats that fall into each category (as well as "neither" if applicable) would be very helpful in displaying the distribution of phenotypes they have characterized.

Secondly, data from cocaine and oxycodone self-administering rats appear to be combined together (as in Figure 1b). As these drugs are of different classes and the addiction index is calculated from different behaviors, it is necessary to show data sets from each drug separately.

Minor:

Abstract:

- Quick text edit to clarify that self-administration is intravenous self-administration, as oxycodone can also be administered orally. This would also be helpful in the Introduction.

Methods:

- Clarification of timeline for terminal samples. Because there are a lot of different timepoints in this study, simply stating "Rats are sacrificed at three different timepoints following the last long access session..." would be beneficial.

- It is unclear if all behavioral testing was completed in the terminal intoxication group due to the necessarily short window before sacrifice. If not, then the addiction index would not include all behaviors for the intoxication group. Please clarify this point.

- Clarification of information for naive rats. Did naive rats self-administer saline and undergo all testing procedures? Currently, the text only states "... or from naive rats". Similarly, please clarify number of samples available from naive rats. Are these numbers included in the totals listed in Table 1?

Results:

- Please state drug doses. They are available in the extended methods but would be helpful to have stated in the results as well.

Figure:

- Please add times from the last long access session to Figure 2 to better display when terminal samples for the 3 groups were taken.

*******************************

Full comments from Reviewer #2:

*******************************

Here the authors describe the creation of a biobank containing numerous samples from rats trained to self-administer intravenous cocaine or oxycodone. The authors include males and females from the genetically diverse Heterogeneous Stock rats, and describe the process by which they characterize individual vulnerability to cocaine or oxycodone "addiction-like" behaviors. This will be a valuable resource for the addiction research community, particularly for those that lack the requisite tools, time, and space to perform long access self-administration experiments.

1) I find the timeline diagram in Fig 1a to be particularly confusing with the symbols for behavioral testing. It would be helpful to detail the actual timelines of the various behavioral tests because, as represented in the figure, it is difficult to ascertain the interval between tests. Since some behavioral characterizations differ between oxy and cocaine (along with the number of ShA training sessions, etc) it may be useful to create a separate timeline figure for each drug.

2) Please clarify if rats are also housed 2/cage after IV catheter implantation.

3) Line 100: Each breeder pair contributes one male and one female to subsequent cocaine and oxycodone cohorts. Does this include drug-naive rats? i.e. are the "age-matched control animals" referenced on line 206 littermates of the drug-exposed animals, or from separate breeding pairs?

4) Please clarify if the self-administration sessions take place during the light or dark cycle, especially since ZT differs between oxy and coc groups.

5)Please revise extended data fig 2-1 caption to indicate the tissue punches are samples from dorsal BNST, not total BNST

6) Line 296: revise to vulnerability and resilience to SUD-like behavior (or addiction-like behavior). Rats don't have substance use disorders.

7)Line 299: I don't see why it is necessary to introduce the concept of personalized medicine when this is a biobank of rat tissue. Perhaps the authors mean to suggest that seq data from their biobank tissues will be a useful comparison for human GWAS and other human samples? Or informing personalized treatment [for humans]? They certainly can't implement personalized treatments for the rats.

## Author Response

Blinded Point-by-point reply to all questions and concerns raised in the review process (attachment 1 of rebuttal letter)

Synthesis of Reviews:

1) The authors should clarify their reporting of the behavioral studies performed on the animals included in the Biobank. Figure 1A was confusing to the reviewers and would benefit from breaking the schematic into separate panels for the different behavioral tests that were run on different cohorts. Since behavioral characterizations also differed between the oxy and cocaine animals (along with the number of ShA training sessions, etc) it would be useful to create separate panels for each drug. Figure 1A may also be more appropriate to combine/integrate with Figure 2 instead of with Figure 1B (see comment 2).

Response: We appreciate this feedback on the figures and apologize that they were not clear before.

Taking into account all this feedback and suggestions, Figure 1 was completely redesigned and merged with Figure 2 (in a new Figure 2). It now shows the sequence of the different behavioral tests and both longitudinal and terminal sampling, with additional details on the experimental protocols for cocaine and oxycodone separately. Moreover, the unclear symbols were replaced by wording to further help interpretability of the timeline.

2) Regarding Figure 1B, it would be helpful if this figure contained a summary of raw data/info on the animals in the Biobank instead of the derived z-score metrics. Researchers are likely to want to know the demographics (age, sex) of animals in the Biobank, as well as levels of lever-pressing and/or amount of drug intake that was achieved by animals in the Biobank, and not just the z-scores of their responding relative to the mean. Reporting variance in drug intake could also assist readers in understanding their ability to obtain data from escalators vs. non-escalators from the Biobank. While some of this information is in Table 1, a graphical summary would be more useful than the present data shown in Figure 1B.

Response: To give the readers more insight in the behavioral responding of the diverse animals with raw data, we now present data on escalation, motivation and compulsivity or hyperalgesia obtained through self-administration under a fixed ratio, progressive ratio and with a contingent foot shock or Von Frey respectively, for one cohort (60 animals) of both the cocaine and oxycodone biobanks in a new Figure 3.

To help the reader understand the difference between animals with high and low addiction-like behaviors, we present the data with their variance following a median split of the addiction index. Additional information on the age of the rats at the different stages of the protocol was added in the legend of Figure 2 and the methods section.

In addition, it appears that data from cocaine and oxycodone self-administering rats were combined together in Figure 1B. As these drugs are of different classes and the addiction index is calculated from different behaviors, it is necessary to show data sets from each drug separately. Finally, the figure should employ a legend for what the light and dark blue bars represent. Breaking this analysis apart from Figure 1A and expanding it into its own figure may be a clearer way to present the information.

Response: The original Figure 1B only represented data from the oxycodone Biobank, we apologize that this was not clear and fully agree with the reviewers that oxycodone and cocaine data should not be combined. As the figures were fully reworked to address the comments above, new raw behavioral data from both the oxycodone and cocaine animals separately is represented in a new Figure 3. We also ensure that the new legend is fully explanatory of the different groups and colors. 3) It is not clear whether “age-matched control animals” (line 206) refer to littermates of the drug-exposed animals, or if these are from separate breeding pairs. Please clarify.2

Response: The age-matched naive control animals are both littermates of the drug-exposed animals or from separate breeding pairs. In fact, all drug-exposed animals (except a male and female sibling pair for the oxycodone and cocaine biobanks each) come from different breeding pairs to maximize genetic diversity for the following GWAS study. The age-matched naive control animals are thus similarly or more closely related to each other and the drug-exposed animals than the drug-exposed animals to themselves. We believe this breeding protocol and rat selection for the two biobanks was not clearly explained in the manuscript. To clarify, the methods section was extended to include some information from the supporting information and additional explanation on the breeding and selection was added.

Under animals: “To maximize genetic diversity, each breeder pair contributes only one male and one female to subsequent cocaine and oxycodone cohorts.” Under the new surgery section: “In each cohort, a number of rats were not surged and subsequently not exposed to drugs. These naive rats are from the same cohort (same or different breeder pairs) as the drug-exposed animals and are thus age-matched control animals.”

4) There were several methodological details that were omitted from this manuscript and should be reported. See full reviewer comments on these below.

Response: The methodological details that were missing were added to the paper, as indicated in detail below. Moreover, the extended materials and methods was moved into the main text’s materials and method’s section, to help clarify the approach.

5) A graphical abstract could enhance this paper.

Response: A graphical abstract was designed according to the guidelines and added in the upload to enhance the paper.

Full comments from Reviewer #1:

This manuscript has the potential to advance the field if more information about the recorded behaviors are provided by the authors in a revision. These collected samples are targeted for use in laboratories that would not otherwise be able to generate similar tissue sets. Additionally, as the authors ran both cocaine and oxycodone IV self-administration to produce comparable biobanks, samples from these biobanks have the potential to confer meaningful data about parallels between the two classes of drugs of abuse.

Response: We thank the reviewer for the positive evaluation of our work and recognizing the value of our standardized behavioral characterization and generated Biobanks However, as presented, this manuscript itself does not provide novel data to advance the field.

Response: We apologize for focusing only on the novelty of the Biobanks as a resource in the first submission and added novel data according to the reviewer’s suggestions below for this resubmission.

Major:

This manuscript introduces two biobanks that aim to expand the accessibility of tissue obtained following rat drug self-administration, which could benefit many researchers. However, as the two data panels (Figure 1b and Figure 3) presented are already published figures, the manuscript does not currently offer any novel data. As the authors have done a significant amount of work characterizing a variety of drug-3 related behaviors, displaying these data would allow for better transparency and provide further validity to the banked samples. There are many approaches that would achieve this point. For example: - Providing data related to average intake or responding across FR1 sessions is important for understanding the level of drug intake that was achieved in these studies. The authors could also replicate known effects, such as sex differences in intake/motivation.

Response: We now present data in a new Figure 3 on escalation for a cohort of cocaine-administering animals and oxycodone-administering animals, which shows the intake achieved over the different sessions under FR1. We additionally provide data on motivation and compulsivity for cocaine administering animals or motivation and hyperalgesia for oxycodone-administering animals obtained through progressive ratio, FR1 with a contingent foot shock or Von Frey. To supplement the statements form the results section, as suggested by the reviewer in his next point, we decided to split up the animals according to Addiction Index into a group with high and low addiction-like behaviors, instead of according to sex. Note that a full paper is in preparation that will include all the cohorts and extended analysis of sex differences, estrus cycle, addiction index, and interaction between variables. The format of the eNeuro Resource manuscript would not be appropriate for such extended analysis. However, we have added a sentence with references in the discussion to highlight the fact that there are known sex difference in cocaine and oxycodone self-administration and that numerous biological and behavioral measures in the biobank (including self-administration) show sex-difference and that samples can be selected based on sex and estrus cycle to facilitate research on sex differences.

"Sex differences in cocaine and oxycodone intake have been identified using this model, as seen in human, with females appearing more vulnerable (Kimbrough et al., 2020; Ramôa et al., 2013; Roth and Carroll, 2004). Behavioral testing for the Biobank confirmed these findings with significant differences in escalation, intake, motivation and compulsivity. Samples can be selected based on sex and estrus cycle to facilitate research into these differences.”

- Another benefit mentioned by the authors is the ability to detect individual differences. Examples of this from the recorded behaviors would greatly underscore the authors’ points. For example, displaying data for escalators vs non-escalators in cocaine and oxycodone groups would supplement the authors’ statements in the Results section.

Response: We really appreciate this suggestion from the reviewer to help illustrate and underscore our point on behavioral diversity in the HS rats. Therefore, we show the raw data added in the new Figure 3 with a median split according to the addiction index, to illustrate the significantly different behaviors of animals from the groups with high or low addiction-like behaviors.

- Additionally, because the authors split rats into low and high addiction-like groups and state that this characterization is a major benefit of their biobank, percentages of rats that fall into each category (as well as “neither” if applicable) would be very helpful in displaying the distribution of phenotypes they have characterized.

Response: While several metrics are possible to characterize the animals based on the acquired behavioral data and the Biobank user’s application, our currently used metric is the Addiction Index. This is an averaged Z-score of relevant addiction-like behaviors for both drugs separately. Animals are then categorized as having high or low addiction-like behavior based on a median split (positive, high versus negative, low values for the Addiction Index). This means that representation in both groups is 50/50. The description of this process is improved, with inclusion of the use of a median split, to make this clear. 4

Secondly, data from cocaine and oxycodone self-administering rats appear to be combined together (as in Figure 1b). As these drugs are of different classes and the addiction index is calculated from different behaviors, it is necessary to show data sets from each drug separately.

Response: As stated above, the interpretation that the data represents a combination of cocaine and oxycodone behavioral measures is a misunderstanding and we apologize for representing the data in such a way that led to this misunderstanding. In the new Figure 3 the results for the cocaine and oxycodone animals are both shown separately.

Minor:

Abstract:

- Quick text edit to clarify that self-administration is intravenous self-administration, as oxycodone can also be administered orally. This would also be helpful in the Introduction.

Response: Self-administration was changed to intravenous self-administration at first mention in the abstract and introduction.

Methods:

- Clarification of timeline for terminal samples. Because there are a lot of different timepoints in this study, simply stating “Rats are sacrificed at three different timepoints following the last long access session...” would be beneficial.

Response: To improve understanding of when the different samples were collected, the description of the terminal samples in the method section was edited as suggested, by adding the statement and reformulating the description of the 3 timepoints: “After the last long access session and completing the behavioral characterization, rats are sacrificed at three different timepoints. 1) Intoxication: euthanasia is performed during the last self-administration session (after ∼2 h of drug access), 2) Acute withdrawal: euthanasia is performed 18 h (for cocaine) and 12 h (for oxycodone) after the end of the last self administration session, 3) Protracted abstinence: euthanasia is performed 4-5 weeks after the last self administration session.

- It is unclear if all behavioral testing was completed in the terminal intoxication group due to the necessarily short window before sacrifice. If not, then the addiction index would not include all behaviors for the intoxication group. Please clarify this point.

Response: We apologize that this point was not clear. All animals go through the entire behavioral characterization protocol before sacrifice. After, the animals undergo one additional session with or without incubation to achieve a state of intoxication, withdrawal or abstinence in which they are sacrificed. The statement included for the previous comment should make this clear. Moreover, by reworking and merging figures 1 and 2 (in a new figure 2), the entire timeline should also be better illustrated and therefore easier to understand.

- Clarification of information for naïve rats. Did naïve rats self-administer saline and undergo all testing procedures? Currently, the text only states “... or from naïve rats”. Similarly, please clarify number of samples available from naïve rats. Are these numbers included in the totals listed in Table 1?

Response: Additional information on the naive rats was included in response to the synthesis of review questions (see above). Naive rats were not surged, and thus did not self-administer drugs. They undergo the testing procedures that do not require the catheter (e.g. Von Frey or bottlebrush test). This is clarified 5 in the new method section in the main text under behavioral testing “Naive rats underwent the behavioral tests that did not require a catheter.”. The number of naive rats is included in the totals listed in Table 1, this and their contribution to the number of samples is clarified in the Table legend: “Samples from naive animals are included in the given number, they represent ∼ 20% of samples.”

Results:

- Please state drug doses. They are available in the extended methods but would be helpful to have stated in the results as well.

Response: The drug doses for cocaine and oxycodone are now stated in the results section too.

Figure:

- Please add times from the last long access session to Figure 2 to better display when terminal samples for the 3 groups were taken.

Response: As stated above, Figure 2 was incorporated into Figure 1 and completely reworked (into a new Figure 2), which helps to better display when terminal samples for the 3 groups were taken.

*******************************

Full comments from Reviewer #2:

*******************************

Here the authors describe the creation of a biobank containing numerous samples from rats trained to self administer intravenous cocaine or oxycodone. The authors include males and females from the genetically diverse Heterogeneous Stock rats, and describe the process by which they characterize individual vulnerability to cocaine or oxycodone “addiction-like” behaviors. This will be a valuable resource for the addiction research community, particularly for those that lack the requisite tools, time, and space to perform long access self-administration experiments.

Response: We thank the reviewer for the positive evaluation of our work and recognizing the value of our standardized behavioral characterization on male and female HS rats and generated Biobanks

1) I find the timeline diagram in Fig 1a to be particularly confusing with the symbols for behavioral testing. It would be helpful to detail the actual timelines of the various behavioral tests because, as represented in the figure, it is difficult to ascertain the interval between tests. Since some behavioral characterizations differ between oxy and cocaine (along with the number of ShA training sessions, etc) it may be useful to create a separate timeline figure for each drug.

Response: We appreciate this feedback on the figure and apologize that this was not clear before. As stated above, Figure 1 was completely redesigned and merged with Figure 2 to make the timeline more clear (in a new Figure 2). It now shows the sequence of the different behavioral tests and additional details for both of the experimental protocols for cocaine and oxycodone.

2) Please clarify if rats are also housed 2/cage after IV catheter implantation.

Response: Rats were indeed group-housed with 2/cage after IV catheter implantation. This was clarified in the methods section under animals, by including the statement “... for the rest of the experiment housed two per cage...”6

3) Line 100: Each breeder pair contributes one male and one female to subsequent cocaine and oxycodone cohorts. Does this include drug-naïve rats? i.e. are the “age-matched control animals” referenced on line 206 littermates of the drug-exposed animals, or from separate breeding pairs? Response: Additional information on the naive rats was included in response to the synthesis of review question 3 (see above).

4) Please clarify if the self-administration sessions take place during the light or dark cycle, especially since ZT differs between oxy and coc groups.

Response: The self-administration sessions (and all other behavioral tests) were performed in the dark cycle of the animals. The different light cycles of the cocaine and oxy animals, as mentioned in the methods section under animals, allowed for running them both during their own dark cycle in the same boxes during the day and night resp. This was clarified by adding a statement in the new material and methods section of the main manuscript under behavioral testing “All behavioral tests were performed in the dark cycle of the animals.”

5) Please revise extended data fig 2-1 caption to indicate the tissue punches are samples from dorsal BNST, not total BNST

Response: The reviewer is correct, the revision has been made as suggested (in what is now figure 1).

6) Line 296: revise to vulnerability and resilience to SUD-like behavior (or addiction-like behavior). Rats don’t have substance use disorders.

Response: The reviewer is right. SUD has been corrected to “SUD-like behavior”.

7)Line 299: I don’t see why it is necessary to introduce the concept of personalized medicine when this is a biobank of rat tissue. Perhaps the authors mean to suggest that seq data from their biobank tissues will be a useful comparison for human GWAS and other human samples? Or informing personalized treatment [for humans]? They certainly can’t implement personalized treatments for the rats.

Response: The reviewer is right here too, obviously our aim is not to develop personalized treatments for the rats, but indeed informing personalized treatments for humans. This could be achieved by comparing the rat GWAS results with human GWAS or by identifying novel markers in rats that have been missed in human GWAS (due to limited number of samples, lack of longitudinal information, lack of controlled environment, trustworthiness of subjective reporting, etc.) that can then be translated to humans. We apologize that this might not have been clear and added “human” to the discussion, to make this clear
